# Reports of Surgical Cases

**Published:** 1860-06

**Authors:** E. Andrews

**Affiliations:** Professor of Surgery in the Medical Department of Lind University; Chicago


### REPORTS OF SURGICAL CASES.

By E. ANDREWS, M. D.,
Professor of Surgery in the Medical Department of Lind University.

*Discoloration of the Crystalline Lens.*—Mr.——, of——, Ill. applied to me for an examination of his eye. He had come in from the country to be treated for blindness, and applied to a notorious advertising eye quack who practices in this city. He paid him fifty dollars in advance, and received in return a valuable promise of relief. Having spent some time under his care without material advantage, he at length got his eyes opened—not to the sunlight, but to the fact that he was in the hands of a deceiver—and was out of money. In this situation he called upon my colleague, Prof. Hollister, who pitying his condition, gave him his own advice freely, and also brought him to the notice of myself and Dr. Holmes. It appeared that the quack had pronounced the case to be amaurosis, and had given a passably good treatment for that disease, but neglected to make a proper internal examination of the eye. On examination we found the organ looking nearly natural, the pupil appearing black, and to the naked eye seeming perfectly clear. The edge of the iris was a little altered by former inflammation, and adherent to the lens so as not to dilate on the application of atropine.

The case had therefore all the appearance superficially of a true amaurosis.

On testing the condition of the retina, we found, however, that the patient could see a candle across a room, and distinguish accurately the position of any bright object, and even sometimes the form of large distinct letters. This test, which is the one adopted by the best authorities for determining the power of the retina in cases of cataract, showed that membrane to be in a good condition, and that the quack diagnosis was a complete error. The opthalmoscope confirmed our opinion, by showing that there was some dark semi-opaque substance in the eye which prevented any clear view of the posterior surface of the organ. On testing the eye by the reflection of a candle, only the anterior image could be produced, showing that the crystalline lens was no longer smooth, and that light was not transmitted clearly through it. In short, it was either a true black cataract, or else a discoloration of the lens by adhesion of pigment from contact with an inflamed and contracted iris.

We advised the patient to discontinue all medication, and whenever he was able to come again to the city, to place himself in competent hands for removal of the cataract by operation.

---

*Fractures of the Skull.* Case 1.—R. W., aged 10 years, was kicked by a horse in the forehead. On my arrival at the house I found him with a wound in the eye-brow, which communicated with a fracture in the outer table of the skull, produced apparently by the cork of the horse's shoe. The fractured portion was a little depressed upon one side, but as there were no symptoms of compression of the brain, and the patient retained all his mental faculties perfectly, I did not interfere with the bone. The skin was simply drawn together with adhesive straps, and left for union. In this patient, although so young, I think the frontal sinuses were so much developed that the fracture was purely in the front wall of the sinus, while the inner table behind those cavities was not injured. The result justified this view, for in ten days the wound was healed, and the child well without any untoward symptoms.

Case 2.—J. A., fell upon a railroad track in front of a hand car. The wheel struck the frontal bone, near the saggital suture, producing a depressed fracture, and the usual coma and stertor, and the other symptoms dependent on compression of the brain. On my arrival, some hours afterwards, I found the patient partly recovering his senses, and the symptoms of compression so far passing off as to render the trephine scarcely necessary, so far as that condition was concerned.

The examination of the injury, however, showed the following condition. The skull was fractured at the point of injury, and the bone comminuted and driven, in a concave form by the blow of the rounded flange of the car wheel. The fragments were impacted together, so as to form an inverted arch capable of obstructing completely the escape of pus, or any necrosed fragments of the inner table, should either result from the injury. I judged it safest therefore to apply the trephine. On penetrating through the external table, the button of bone thus formed separated from the inner table and came away alone. The inner table was found extensively splintered, and separated both from the outer table and the dura mater. One of the splinters had penetrated to the brain, allowing a small quantity of the gray matter to ooze out. After elevating the firmer parts of the depression and removing the loose pieces, the scalp was drawn over the part, and a cold water dressing applied. The other injuries of the patient now claimed attention. A great number of small cuts, scratches and bruises existed, but the chief trouble was a double fracture of the jaw. The lower maxilla was broken through on both sides, and the central portion thus deprived of support was retracted by the action of genio-hyoid muscles, and could not be retained in its natural position by the ordinary dressings. I therefore called in a dentist, who took an impression of the inside of the mouth in plaster of paris, by means of which he moulded a silver plate to fit over the arch of the teeth. This being done, the lower jaw was strapped up against the upper, and made to retain its form perfectly. The patient, of course, was fed upon liquid food.

On the second day, I was alarmed to perceive evidences of a decided suppurative diathesis. Every scratch upon the body began to exude pus. I administered equal parts of muriatic acid and muriated tincture of iron in doses of 30 drops every hour. In twenty-four hours more I was relieved to find the suppurative tendency perfectly controlled, and the appearance of all the wounds much healthier. From this time onward the progress was favorable, and the patient made a complete and rapid recovery.

Case 3.—This patient received a blow on the head from some object at a railroad accident. The upper part of one parietal bone was fractured and depressed. The case presented no unusual phenomena; the trephine and elevator were applied and a good recovery followed.

---

*Injuries Simulating Fractures of the Skull.* Case 1. A man was thrown from a wagon and taken up in an insensible condition. I had him conveyed to his house and there made an examination. The patient was comatose, but the pulse not as slow as in most cases of compression. On the occipital bone there was a distinct depression, with one well defined edge, but no injury to the scalp. After careful examination, I decided to await further developments, as the depression did not present all the appearances of a recent injury. After two hours the patient was perfectly conscious; the depressed spot was not sore, nor did it present any appearance of violence. In short, it proved to be an accidental irregularity, either congenital, or else the result of some old injury. The man made a good recovery.

Case 2. A boy, aged 12 years was thrown from a wagon. On examination I found the scalp separated from a portion of the occipital bone, but not torn. The separated patch was distended with blood, making a soft fluctuating tumor. At the lower part of the tumor, at the insertion of the trapezius muscle, there was a blunt edge to be felt with an apparent cavity above it. The patient was comatose, but the breathing was not stertorous, nor did the pulse indicate compression. I opened the tumor, evacuated the blood, and explored the cavity with my finger. The skull was perfectly sound, and the prominent ridge which I felt at first proved to be the upper end of the trapezius muscle, which was torn from its insertion and presented a sort of sharp edge under the skin. The patient was more or less impaired in his mental functions for several months after the injury, but finally recovered.

